# A Metastatic Invasive Mole in a Perimenopausal Woman: A Rare Case

**DOI:** 10.7759/cureus.40121

**Published:** 2023-06-08

**Authors:** Ana I Tomé, Rita Palma, Sofia Carralas Antunes, Madalena A Tavares, Elisa Pereira

**Affiliations:** 1 Gynecology, Hospital Garcia de Orta, Almada, PRT; 2 Pathology, Hospital Garcia de Orta, Almada, PRT; 3 Gynecology, Hospital Vila Franca de Xira, Vila Franca de Xira, PRT; 4 Gynecologic Oncology, Hospital Garcia de Orta, Almada, PRT

**Keywords:** uterine artery embolization, systemic chemotherapy, hydatidiform mole, perimenopausal, invasive mole, gestational trophoblastic disease (gtd)

## Abstract

Gestational trophoblastic neoplasia (GTN) represents a heterogeneous group of pregnancy-related tumors that usually develop from the malignant transformation of trophoblastic tissue after molar evacuation. The first presentation as an invasive mole is particularly rare. GTN is considered the most curable gynecological malignancy as most cases are treated successfully with chemotherapy agents. Although extremes of reproductive age are an established risk factor for complete moles, GTN is extremely rare in perimenopausal women. GTN should be considered in the differential diagnosis of patients with abnormal uterine bleeding. Delays in the diagnosis and treatment can worsen the prognosis of patients with GTN.

Here, we describe the case of a 54-year-old woman who presented to the emergency department with abdominal pain and heavy vaginal bleeding. She reported pregnancy-related symptoms that had developed over two months but was apprehensive to search for medical care. The final diagnosis was an invasive mole that had a catastrophic clinical course. Arterial embolization should be considered in patients with uncontrollable vaginal bleeding and hemodynamic instability.

## Introduction

Gestational trophoblastic neoplasia (GTN) includes a heterogeneous group of malignant pathologies with distinct pathogenesis, morphological characteristics, and clinical presentation which can arise after any type of pregnancy [1]. Invasive hydatidiform mole is the most frequent form of GTN, defined histologically by myometrial and/or lymphovascular invasion by trophoblast cells encompassing molar villi or by the presence of metastases containing molar villi [2]. Other rarer forms include choriocarcinoma, placental site trophoblastic tumor, and epithelioid trophoblastic tumor [1-3].

Invasive mole occurs more frequently after a previous diagnosis of hydatidiform mole as the initial presentation as an invasive mole is particularly rare. Despite the reported regional variations, the incidence of a hydatidiform mole varies between 0.57 and 2 per 1,000 pregnancies. [3] Postmolar GTN develops in 15-20% of cases of complete moles and 1-5% of partial moles [2].

The clinical presentation of invasive mole includes nonspecific symptoms such as abnormal uterine bleeding, enlarged uterus, pelvic pain, and effects associated with stimulation by human chorionic gonadotropin (hCG) [3,4]. Uterine perforation, hemoperitoneum, or profuse bleeding are extremely rare [5]. Metastasis occurs in approximately 15% of invasive moles most often in the lungs, vagina, liver, and brain [2,4].

Although GTNs are tumors with high local invasive and metastatic potential, they are curable in 90-100% of cases [3]. As it is a rare disease, in which an appropriate approach has a prognostic impact, patients with GTN should be guided by a multidisciplinary team in reference centers.

## Case presentation

A 54-year-old perimenopausal woman, with an unremarkable past medical history, presented to the emergency department with complaints of abdominal pain and profuse vaginal bleeding. She also reported pregnancy-related symptoms such as abdominal distension, nausea, and breast tenderness for the past two months but a negative urine pregnancy test. Considering the obstetrical history, the patient had eight pregnancies, with two uncomplicated spontaneous abortions, five normal vaginal deliveries, and one cesarean section. Her last pregnancy had been eight years ago. The patient had a regular menstrual bleeding pattern until two months before the onset of her symptoms. She reported being sexually active and denied the use of contraception.

**Figure 1:**
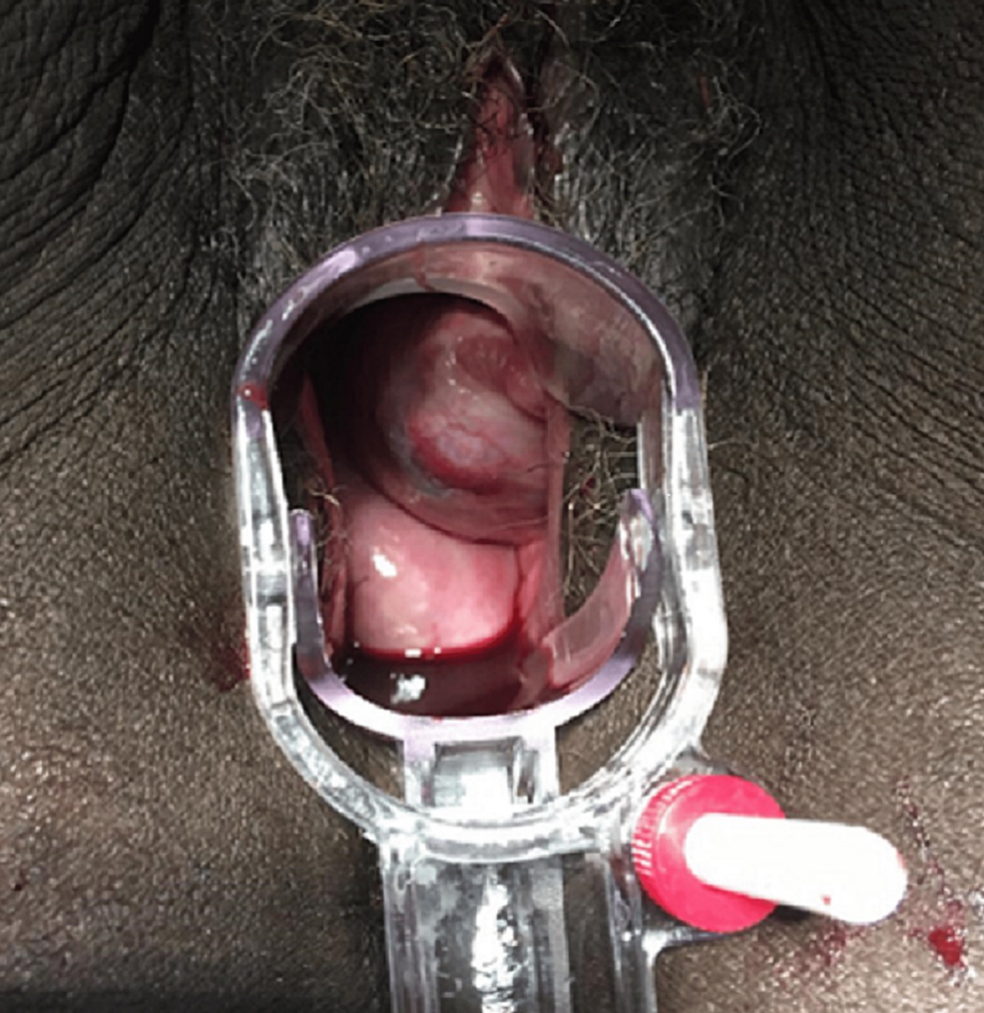
Speculum examination showing a friable 7 cm soft tumor in the anterior and left wall of the inferior vagina.

On admission, she was tachycardic with normal blood pressure. On gynecological examination, a solid and painful mass extending 5 cm above the umbilicus was palpated, compatible with a 30-week pregnancy. Speculum examination revealed a 7 cm soft tumor in the anterior and left wall of the inferior vagina (Figure 1).

**Figure 2:**
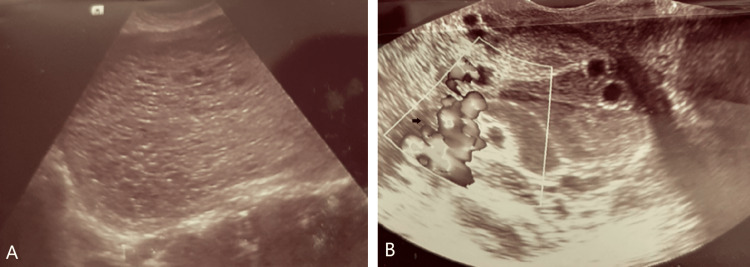
Transvaginal ultrasonography image. (A) Heterogenic and irregular ill-defined mass lesion invading the uterus. (B) Color Doppler sonography of the hypervascularized vaginal lesion.

The external cervical os was closed with no macroscopic lesions identified despite the moderate vaginal bleeding at the time of examination. Ultrasonography revealed a very enlarged uterus (impossible to measure with both transvaginal and transabdominal transducer) with a heterogeneous vesicular mass that obliterated the endometrial cavity (Figure 2A). Adnexa was not identified. On transvaginal ultrasound, a heterogeneous hypoechoic mass measuring 46 × 36 mm, highly vascularized, was identified on the lower vagina (Figure 2B).

**Figure 3:**
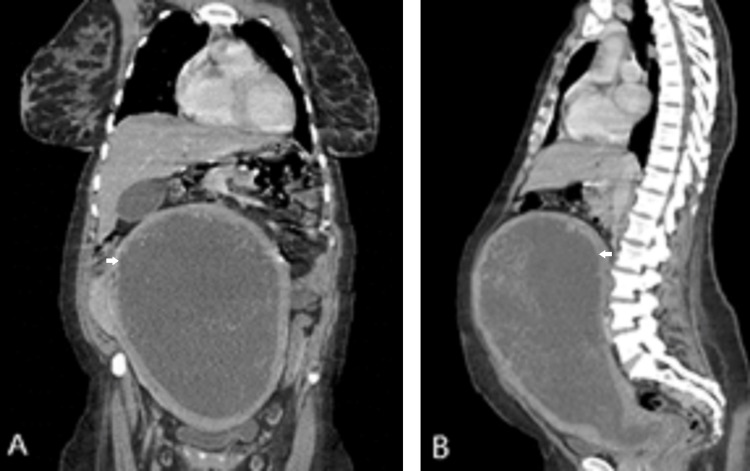
(A, B) Computed tomography scan of the abdomen and pelvis showing an enlarged uterus with heterogeneous highly vascularized content. (A) Coronal view. (B) Sagittal view.

Blood tests revealed anemia with a hemoglobin level of 9.3 g/dL and elevated serum creatinine (1.0 mg/dL at admission, with an elevation to 2.0 mg/dL two days after admission), with normal coagulation tests and normal hepatic function. Serum hCG was very high (>1,000,000 IU/L) with abnormal thyroid function (thyroid-stimulating hormone (TSH) <0.01 mIU/L, free thyroxine (T4) 2.89 ng/dL, and free triiodothyronine (T3) 7.35 pg/mL). A thoraco-abdomino-pelvic computed tomography (CT) revealed an enlarged uterus measuring 28 × 19 × 13 cm filled with highly vascularized heterogeneous content, suggestive of a complete hydatidiform mole (Figures 3A, 3B) In the perineum, near the anterior vaginal wall, there was a vaginal nodule compatible with a metastatic lesion with a vascular pedicle in continuity with the underlying intrauterine mass. Multiple nodular lesions were also identified in both basal lung segments, with the largest measuring 7 mm. An endometrial biopsy was performed.

**Figure 4:**
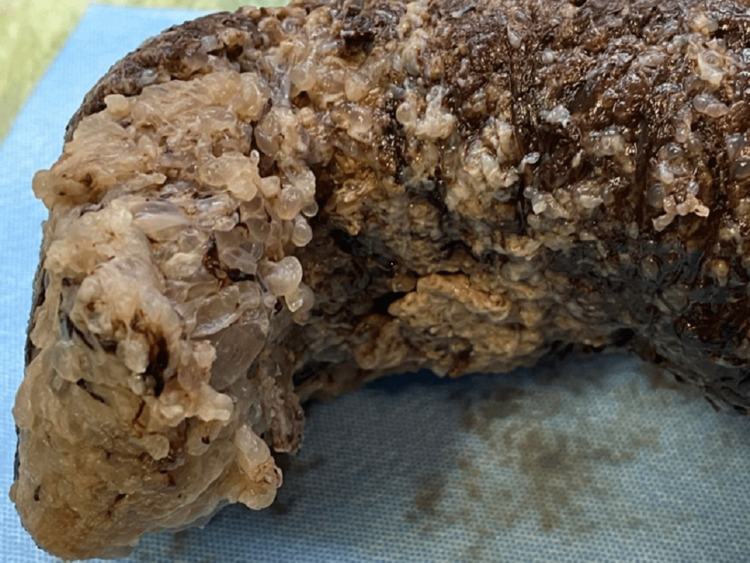
Vesicular tissue expelled from the uterus on day two after admission. Bloody and brown tissue fragment, partially necrotic, weighing 767 g and measuring 19 × 7.5 × 9 cm, with semi-transparent vesicles of variable sizes on one of its sides.

Two days after admission the patient experienced an acute episode of abdominal pain, and a large amount of vesicular tissue was expelled from the uterus, followed by heavy vaginal bleeding (Figure 4).

**Figure 5:**
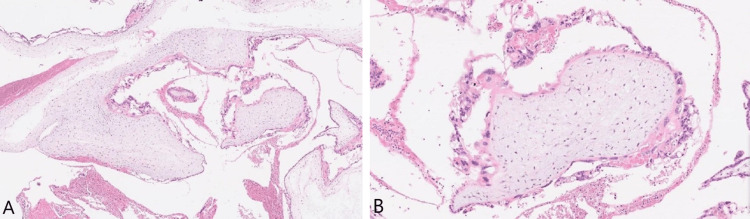
(A) Hematoxylin and eosin stain shows diffuse enlargement of villi with marked hydropic changes and occasional cistern formation. (B) Villi are lined by hyperplastic trophoblast, occasionally in a circumferential pattern, with remarkable cytologic atypia.

Pathological examination of the vesicular mass revealed a complete hydatidiform mole not being able to exclude invasive mole due to the lack of myometrial tissue and blood vessel invasion in the sample (Figures 5A, 5B). Considering the radiological evidence of lung metastases, the patient was clinically diagnosed with a complete hydatidiform invasive mole.

**Figure 6:**
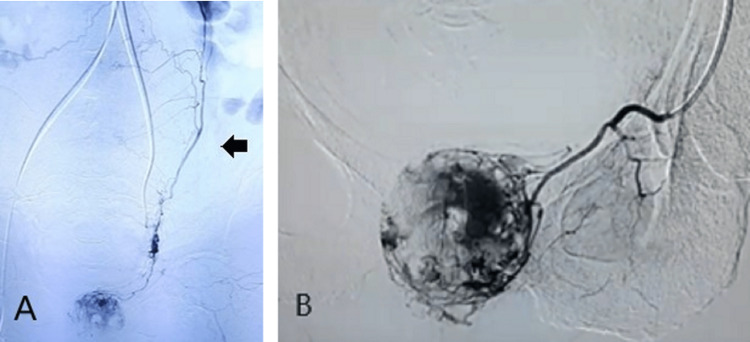
Angiography image obtained before uterine artery embolization. (A) Selective left uterine angiography showing a hypertrophied left uterine artery. (B) Pelvic hypervascular mass in the medium part of the vagina, vaginal metastasis.

Due to clinical deterioration and the risk of uncontrollable vaginal bleeding, the patient underwent an angiography which showed dilated uterine arteries as well as a pelvic hypervascular (vaginal metastasis) mass in the medium part of the vagina supplied by three major branches (a cervicovaginal branch of the left uterine artery and a left aberrant obturator artery arising from the inferior epigastric artery and by the right obturator artery). Both uterine arteries and the three main branches of the vaginal metastasis were embolized. The embolization procedure effectively controlled the vaginal bleeding, and the patient could start chemotherapy (Figures 6A, 6B).

According to the International Federation of Gynecology and Obstetrics (FIGO) staging system, the patient was at stage III and had a high-risk score (score 8) considering the prognosis scoring system for the GTD of the World Health Organization (WHO). A combination chemotherapy regimen with etoposide, methotrexate, actinomycin-D, cyclophosphamide, and vincristine was initiated. Two days after the first chemotherapy cycle, the treatment was suspended due to severe pancytopenia and profuse uterine bleeding, and a massive transfusion protocol was initiated followed by uterine tamponade packing. In this context, the patient was admitted to the intensive care unit and eventually died two days after due to multiple organ dysfunction.

## Discussion

GTN is considered a highly curable group of gynecological malignancies. Most GTNs, particularly invasive moles and choriocarcinomas, are highly chemosensitive tumors with overall survival cure rates as high as 95% in the case of patients with high-risk tumors [3,6]. In fact, GTNs are particularly highly vascularized tumors due to the proliferation of trophoblastic tissue and invasion of the myometrium and adjacent structures that favor the development of multiple fragile vessels and, in rare instances, arteriovenous malformations [7]. Diagnosis of invasive moles is usually based on hCG level changes (with rising or plateau levels) during the follow-up after molar evacuation. Rarely, the diagnosis is made after a spontaneous abortion, an ectopic pregnancy, or a term pregnancy [3]. In these cases, the most common presentation is abnormal vaginal bleeding, enlarged uterus, and abdominal tenderness. However, an invasive mole may present with life-threatening complications, including heavy bleeding, uterine perforation, hemoperitoneum, and acute abdominal pain [5]. We present an unusual clinical course of a perimenopausal woman who had the final diagnosis of metastatic invasive mole. She presented to the emergency department in a relatively unstable condition with complaints of profuse vaginal bleeding and abdominal pain. Despite the extremely enlarged uterus, there were no signs of uterine perforation or hemoperitoneum. A 7 cm vaginal metastasis was also found upon examination. This lesion had a vascular pedicle communicating with the uterine mass that significantly increased the risk of bleeding in this case. The diagnosis of an invasive mole was assumed after an anatomopathological examination of the aspirated and expelled uterine tissue and radiological evidence of pulmonary and vaginal metastasis.

In this case, it is difficult to establish the type of pregnancy that led to the progression to an invasive mole as there was no history of mole pregnancy, and the patient had a term cesarean section eight years before. She had a regular menstrual pattern until two months before the initial symptoms. Consequently, the disease most likely began as an undiagnosed complete mole that progressed to an invasive mole within two months. Although spontaneous pregnancy is rare in perimenopausal women, extremes of maternal age are an established risk factor for complete molar pregnancies. Abnormal gametogenesis and fertilization of the ovum is the probable mechanism of disease development [3-5]. This case demonstrates the importance of considering GTN in cases of abnormal uterine bleeding in perimenopausal women. Moreover, the promotion of adequate contraception is crucial in this stage of reproductive life [8].

According to the FIGO staging system, our patient had a stage III disease considering the presence of lung metastasis on the CT scan. Based on prognostic WHO factors, she had a score 8 disease (GTN stage III:8). A risk score above 6 is considered high-risk GTN, and these patients should be treated with multiple-agent chemotherapy regimens as first-line therapy [3]. Nevertheless, patients should be clinically stable to initiate treatment. In this case, the patient started the first chemotherapy cycle only after she was hemodynamically stable and with an optimized renal function. Regarding the patient’s age and her hemodynamic instability at the initial presentation, a total abdominal hysterectomy was considered, but the development of neovascularization could have led to massive intraoperative blood loss. Considering the suspicion of a communicating vessel between the uterine lesion and the vaginal metastasis, selective arterial embolization was then considered to control the vaginal hemorrhage. After the procedure, the patient could finally be stabilized and started chemotherapy. This case is in line with studies confirming selective embolization of uterine arteries and vaginal metastases as a safe and effective treatment alternative to control bleeding associated with GTN. Nevertheless, there is still concern about whether embolization affects the effectiveness of systemic chemotherapy, although some studies demonstrate comparable oncological outcomes [7,9]. In our case, the patient had a catastrophic outcome with fast deterioration after the first chemotherapy cycle. Chemotherapy could have caused the collapse of the tumor with myelosuppression, septicemia, and multiple organ failure, as reported by some authors [3]. A combination chemotherapy regimen with EMA-CO is currently the first-line therapy for high-risk GTN with cure rates as high as 95%. However, other regimens could be associated with less acute toxicity. Considering the low incidence of GTN, clinical trials evaluating other combination therapies are scarce [10]. Some studies suggest that immunotherapies could be helpful in a subgroup of patients reducing the toxicity of high-dose chemotherapy [3]. In this case, the diagnosis of GTN was established relatively late due to pandemic restrictions and fear of seeking medical assistance. Adverse features that could have predicted the poor outcome of the patient were the delay in the initiation of diagnosis and treatment, the advanced age of the patient, the large tumor size, and the extremely high hCG concentration.

## Conclusions

This case describes the catastrophic clinical course of a perimenopausal woman who presented to the emergency department with profuse vaginal bleeding with the final diagnosis of metastatic invasive mole.

Although rare, GTN should be considered in the differential diagnosis of women with abnormal menstrual bleeding, especially in the perimenopausal period.

Centralized care coordinated by a multidisciplinary team of gynecology, gynecological oncology, medical oncology, radiology, and pathology will promote coordinated treatment plans on a case-by-case basis. Selective arterial embolization is an alternative procedure to an abdominal hysterectomy to manage patients with heavy bleeding resulting in comparable oncologic outcomes.

Prompt recognition of gestational trophoblastic disease is crucial for optimal management with an impact on the patient’s prognosis.
